# Fragile lifespan expansion by dietary mitohormesis in *C. elegans*

**DOI:** 10.18632/aging.100863

**Published:** 2016-01-13

**Authors:** Arnaud Tauffenberger, Alexandra Vaccaro, J. Alex Parker

**Affiliations:** ^1^ CRCHUM, Montréal, Québec, Canada; ^2^ Département de Pathologie et Biologie Cellulaire, Université de Montréal, Montréal, Québec, Canada; ^3^ Département de Neurosciences, Université de Montréal, Montréal, Québec, Canada

**Keywords:** C. elegans, glucose, aging, mitochondria, unfolded protein response

## Abstract

Mitochondrial function is central to longevity and an imbalance in mitonuclear protein homeostasis activates a protective response called the mitochondrial unfolded protein response (UPR^mt^). Toxic compounds damaging mitochondria trigger the UPR^mt^, but at sublethal doses these insults extend lifespan in simple animals like *C. elegans*. Mitochondria are the main energy suppliers in eukaryotes, but it is not known if diet influences the UPR^mt^. High dietary glucose reduces lifespan in worms, and we show that high dietary glucose activates the UPR^mt^ to protect against lifespan reduction. While lifelong exposure to glucose reduces lifespan, glucose exposure restricted to developing animals extends lifespan and requires the UPR^mt^. However, this lifespan extension is abolished by further mitochondrial stress in adult animals. We demonstrate that dietary conditions regulate mitochondrial homeostasis, where induction of the UPR^mt^ during development extends lifespan, but prolonged activation into adulthood reduces lifespan.

## INTRODUCTION

Mitochondria are the powerhouses of the cell, and a consequence of their energy production is the generation of reactive oxygen species (ROS) that may contribute to cellular damage, protein misfolding and ultimately aging [1,2]. Accompanying aging is a decline in the cell's capacity to mitigate damage from ROS and maintain protein homeostasis. Threats to mitochondrial protein homeostasis are reduced in part due to the activity of chaperones like HSP-6 and HSP-60 that are known to combat age-related dysfunction as part of the mitochondrial unfolded protein response (UPR^mt^) [3]. The UPR^mt^ has received considerable attention in recent years as it has been revealed that its role may not be limited to stress response but may also regulate longevity [1,4]. Although the UPR^mt^ is activated against damaging environmental conditions and toxins, it is not known if an organism's dietary status contributes to mitochondrial surveillance mechanisms. We previously reported that *C. elegans* subjected to high dietary glucose were protected against proteotoxicity and environmental stress [5], but had shortened lifespans as previously observed [6]. We wondered whether or not the UPR^mt^ had a role in mediating longevity phenotypes associated with high dietary glucose.

**Figure 1:**
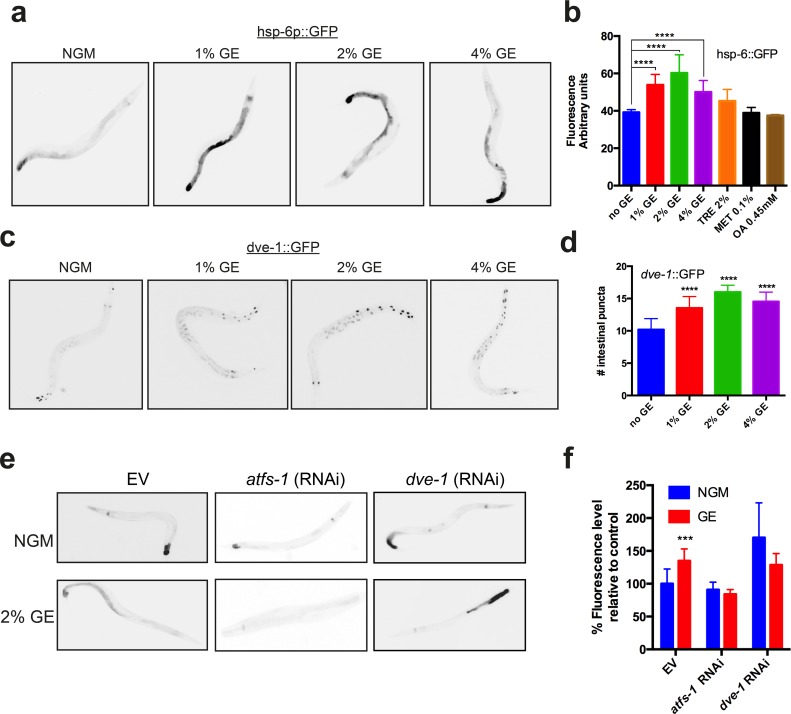
Glucose enrichment induces HSP-6 and DVE-1 expression (**a**) Glucose enrichment (GE) induces expression of a hsp-6::GFP transcriptional reporter at concentrations of 1, 2 and 4%. (**b**) Quantification of hsp-6p::GFP expression (mean ± SD, ****P<0.0001 compared to control). (**c)** GE increases expression of a dve-1::GFP translational reporter at all glucose concentrations tested. (**d**) Quantitative representation of DVE-1 expression after GE treatment (mean ± SD, ****P<0.0001 compared to control). (**e**) Knockdown of *atfs-1* or *dve-1* abolishes GE mediated hsp-6p::GFP expression. (**f**) Quantification of hsp-6p::GFP expression after *atfs-1* or *dve-1* (RNAi) (mean ± SD, ****P<0.0001).

The mitochondrial chaperone reporters *hsp-6p::GFP* and *hsp-60p::GFP* show increased expression in *C. elegans* intestinal tissue as part of the UPR^mt^ [3]. Cultivation of worms under high dietary glucose conditions led to a dose-dependent increase in the expression of *hsp-6p::GFP* (Fig. 1a), but no change was observed for *hsp-60p::GFP* (Supp. Fig. 1). Induction of *hsp-6p::GFP* expression was specific to glucose, as other dietary compounds including trehalose, methionine or oleic-acid had no effect (Fig. 1b). In *C. elegans* the transcription factors ATFS-1 [7] and DVE-1 [8] are required for induction of the UPR^mt^, as is the ubiquitin-like protein UBL-5. We turned to well characterized translational reporters for *dve-1* and *ubl-5* [1,3,8] to investigate the possible role of dietary glucose in activation of the UPR^mt^. We observed that high dietary glucose increased expression of the *dve-1p::dve-1::GFP* reporter (Fig. 1c-d) but did not affect the expression of *ubl-5p::ubl-5::GFP* (Supp. Fig. 1). We next investigated if the transcription factors *atfs-1* or *dve-1* were functionally required for UPR^mt^ induction. RNAi mediated knockdown of either *atfs-1* or *dve-1* abolished the induction of *hsp-6p::GFP* by high dietary glucose (Fig. 1e-f). These data suggest that a specific subset of the UPR^mt^ acts to mitigate potential cytotoxic effects from dietary sources.

## RESULTS

### The UPR^mt^ declines during aging

**Figure 2:**
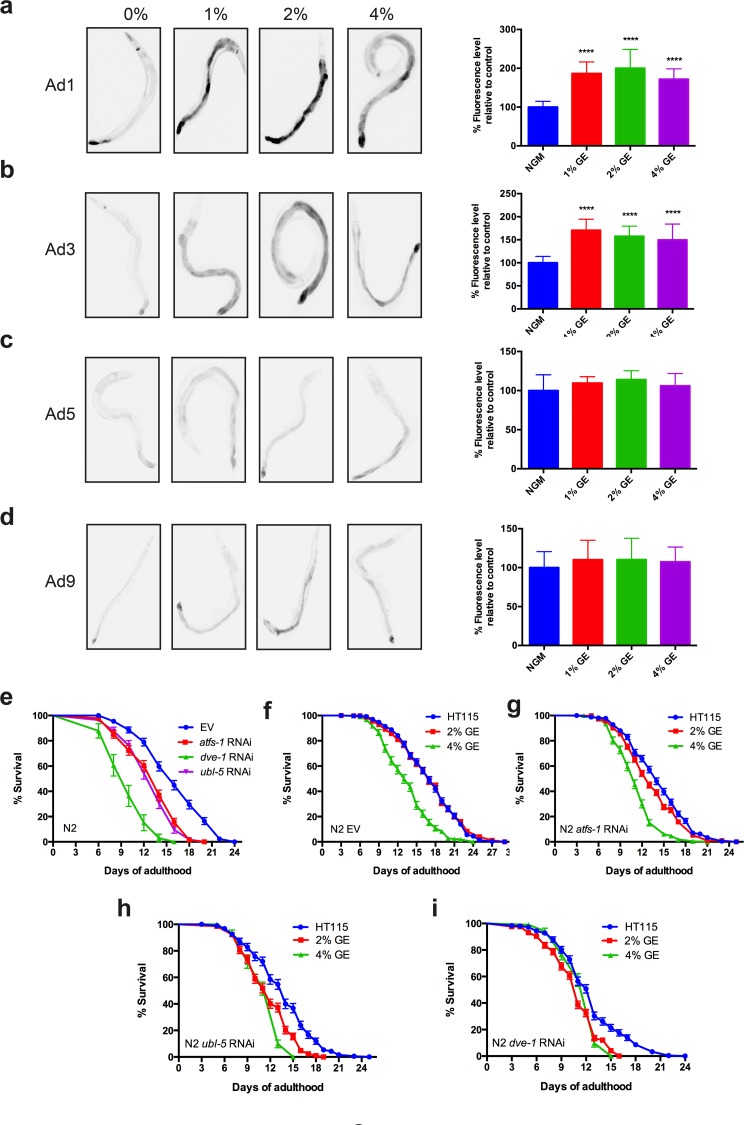
Glucose enrichment maintains HSP-6 expression over time (**a-b**) Photographs and quantification of increased hsp-6p::GFP expression in strains exposed to 1, 2 or 4% GE at days 1 and 3 of adulthood (****P<0.0001, mean ± SD). (**c-d**) GE failed induced expression of the hsp-6::GFP reporter in worms at days 5 or 9 of adulthood (mean ± SD). (**e)** RNAi against *atfs-1*, *dve-1*, or *ubl-5* reduced the lifespan of N2 worms compared to controls (P<0.0001 compared to empty vector (EV) controls). (**f**) Worms grown on EV had reduced lifespan only at 4% GE (P<0.0001 compared to controls). Animals grown on (**g**) *atfs-1*, (**h**) *dve-1* or (**i**) *ubl-5* RNAi had reduced lifespan at 2% and 4% GE (P<0.0001 compared to EV control).

The induction of *hsp-6p::GFP* by high dietary glucose was observed in young adult animals (Fig. 1a). It has been hypothesized that aging may occur partly from an age-related decline of protein homeostasis mechanisms [9], thus we wondered if the induction of *hsp-6p::GFP* by glucose was maintained in aging animals. We observed that exposure to high dietary glucose at three concentrations induced the expression of *hsp-6p::GFP* in animals at days 1 and 3 (Fig. 2a, b) of adulthood, but this induction was lost in animals at days 5 or 9 of adulthood (Fig. 2c, d). To inspect the morphology of the mitochondrial network we used transgenic worms expressing a mitochondrial targeted signal in muscles cells [3]. Normally the mitochondrial network appears as tubular, parallel structures in muscle tissue [1,10].

We observed that these structures became increasingly disordered when the worms were exposed to high dietary glucose as well as during aging (Supp. Fig. 2). These data suggest that the ability of the UPR^mt^ to respond to cellular toxicity decreases during aging and mitochondrial health is damaged by high dietary glucose.

### The UPR^mt^ protects against glucose-mediated lifespan reduction

We next investigated whether the UPR^mt^ protects against glucose-induced reduction of lifespan in adult animals. Wild type N2 animals exposed to RNAi against *atfs-1*, *dve-1*, or *ubl-5* all showed decreased lifespan suggesting that a fully-functional UPR^mt^ is necessary for promoting longevity (Fig 2e). Wild type N2 worms exposed to 2% glucose had lifespan similar to untreated controls, but exposure to 4% glucose reduced lifespan (Fig 2f). Interestingly, lifespan was reduced in worms exposed to either 2% or 4% glucose and treated with *atfs-1*, *dve-1* or *ubl-5* (RNAi) (Fig 2g-i). These data demonstrate that 2% glucose becomes toxic and reduces lifespan in the absence of a functional UPR^mt^, thus the UPR^mt^ may protect organisms against cytotoxicity from dietary sources.

### Early developmental exposure to glucose extends lifespan

**Figure 3:**
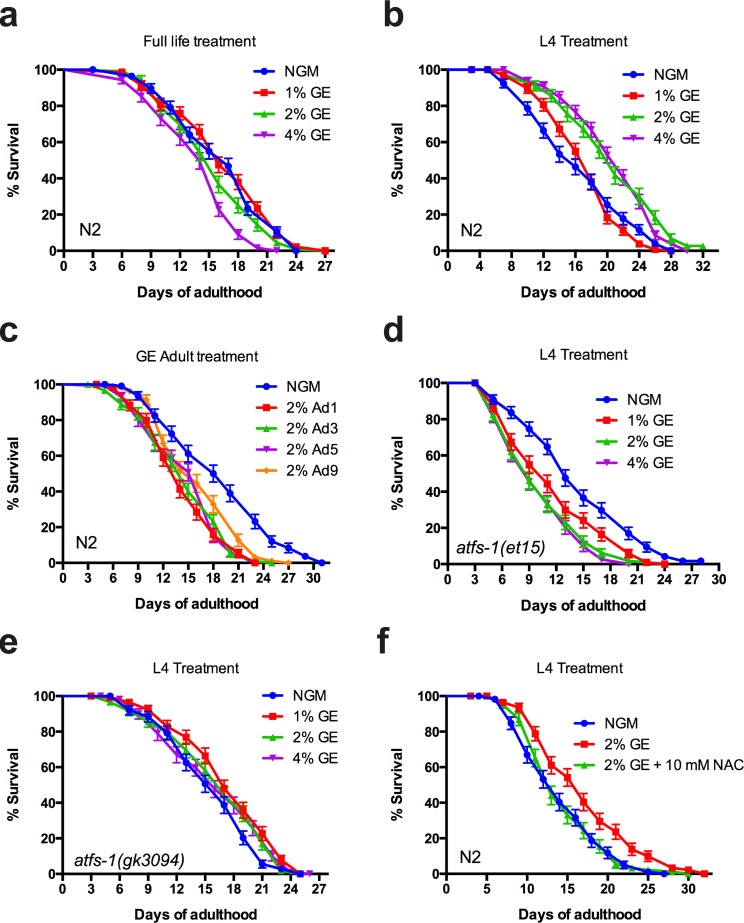
Early or late glucose enrichment has opposite effects on lifespan (**a**) GE reduced lifespan of N2 animals at 4% concentration compared to untreated animals (P<0.0001 compared to control). (**b**) Larval exposure to glucose extends lifespan of N2 animals at concentrations of 2% or 4% compared to untreated animals (P<0.0001 compared to controls). (**c**) Adult exposure to glucose reduces lifespan of wild-type animals (P<0.0001 for all conditions compared to control). (**d-e**) GE failed to extend lifespan in *atfs-1(et15)* and *atfs-1(gk3094)* animals when treatment ceased at the L4 larval stage. (**f**) GE failed to extends lifespan in N2 animals when treated with 10 mM N-acetyl cysteine.

The induction of *hsp-6p::GFP* by glucose does not indicate if the UPR^mt^ is activated to mitigate the toxic effects of glucose, or instead mediates the protective effects of high dietary glucose. Activation of the UPR^mt^ has been observed after inhibition of mitochondrial DNA (mtDNA) translation, or mtDNA depletion and is involved in longevity phenotypes [1]. Furthermore, the loss of UPR^mt^ results in increased susceptibility to stress, while prolonged induction reduces lifespan (*12*-*14*). To delineate the role of the UPR^mt^ in glucose-mediated aging phenotypes, we investigated whether activation of the UPR^mt^ by high dietary glucose could be the cause of glucose-dependent lifespan reduction. Wild-type animals exposed to high dietary glucose for their entire life had reduced lifespan compared to untreated animals, in agreement with previous findings by us [5] and others [6] (Fig 3a). Surprisingly, animals exposed to glucose (2% and 4%) only until the pre-adult L4 larval stage, showed the opposite effect with significant lifespan extension (Fig. 3b). Furthermore, exposing worms to high dietary glucose only during adulthood reduced lifespan compared to control animals (Fig. 3c), suggesting a lifespan-promoting role of glucose during development that becomes deleterious at later adult stages. To determine if the augmented lifespan effect of early glucose exposure was dependent on the UPR^mt^, we investigated the contribution of the transcription factor ATFS-1, which is a master regulator of the UPR^mt^ [7]. We exposed *afts-1(gk3094)* and *atfs-1(et15)* mutants to high dietary glucose from hatching until the L4 larval stage and observed that the enhanced longevity phenotype was lost (Fig. 3d,e). These data suggest that endogenous UPR^mt^ activity via ATFS-1 is required for regulating glucose-dependent lifespan phenotypes.

### Dietary induced mitohormesis extends lifespan

Mitochondria are the major ROS producers at the cellular level and it has long been proposed that ROS molecules and by-products are responsible for organismal aging (*15*). Recent work suggested that ROS rather acts as a signalling molecule with important roles in regulating longevity [11,12]. This phenomenon is known as mitohormesis [13] and the protective effects are lost in the presence of antioxidant compounds. Thus we wondered if the lifespan enhancing effect from early glucose exposure utilized a mitohormetic mechanism requiring ROS. We observed that animals treated with 10 mM N-acetyl cysteine did not have increased lifespan when exposed to high dietary glucose until the L4 stage GE (Fig. 3f). These data suggest a role for oxidative stress in glucose mediated lifespan extension when applied at early, pre-adulthood stages.

### Lifespan extension by glucose requires a functional electron transport chain

Mutations that disrupt mitochondrial function can result in severe pathologies, and mitochondrial dysfunction is linked to multiple neurodegenerative disorders in humans (*19*-*21*). Surprisingly, electron transport chain (ETC) dysfunction can extend lifespan not only in *C. elegans* but also in Drosophila and mice [14]. Thus, we wondered if ETC function was required for the temporal effects on lifespan from high dietary glucose. We observed that the ETC complex II mutant *mev-1(kn1)* and the complex III mutant *isp-1(qm150)* strains showed no increase in lifespan when exposed to high dietary glucose until the L4 larval stage (Supp. Fig. 3). These data suggest that functional mitochondria are required for the increased lifespan from early exposure to high dietary glucose. This is consistent with the previous studies demonstrating that lifespan extending interventions can only be produced at late larval stages (L3/L4) when mitochondrial biogenesis is at it's peak [15].

### Activation of the UPR^mt^ during adulthood abolishes lifespan extension

Glucose treatment over the life of the worm is known to reduce lifespan and in this study we have shown that it is linked to increased *hsp-6* expression. This suggests that activation of the UPR^mt^ during adulthood may reduce lifespan. We wondered if early exposure only might have a similar pattern of *hsp-6* expression.

**Figure 4:**
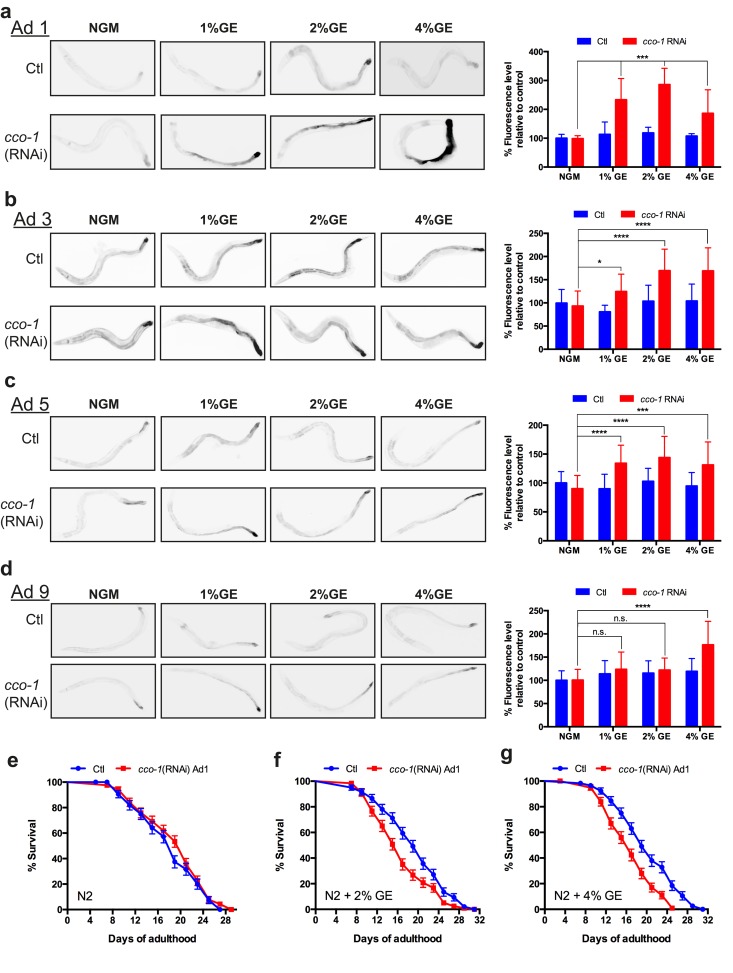
Early exposure to glucose sensitizes nematodes to UPR^mt^ activation (**a-d**) Early exposure (L4) to glucose at different concentrations (1, 2 and 4%) had no effect on *hsp-6* expression during adulthood compared to control NGM conditions. RNAi against *cco-1* was able to induce hsp-6 expression in animals treated at all glucose concentrations until day 5 of adulthood, and only at 2% and 4% at day 9 (mean ± SD., *P<0.05, **P<0.01 and ****P<0.0001 compared to control). (**e**) The lifespan on N2 worms treated with *cco-1*(RNAi) during adulthood was similar to N2 control worms. The lifespans of N2 worms grown on (**f**) 2% or **g**. 4% GE plates was reduced when treated with adult-stage specific *cco-1*(RNAi) compared to worms treated with EV (P<0.01 for 2% GE + *cco-1*(RNAi) and P<0.0001 for 4% GE + *cco-1*(RNAi) versus controls.)

Worms were treated with glucose during larval development and then grown on control NGM. At different aging stages, the animals were transferred to cytochrome c-oxydase *cco-1*(RNAi) plates for a 12h period. *cco-1*(RNAi) is known to induce the UPR^mt^ by disrupting ETC function, and knockdown of this gene during development has been reported to mimic ETC dysfunction with similar longevity phenotypes to knockdown of other ETC genes [16]. Worms grown on NGM and placed on *cco-1* RNAi as adults showed no *hsp-6*::GFP increased expression, consistent with the temporal activation of the UPR^mt^ [17] (Fig. 4). Similar to untreated animals, nematodes exposed to dietary glucose early in life showed no increase in hsp-6::GFP expression. However, animals treated with glucose during development plus adult specific *cco-1* RNAi had increased *hsp-6*::GFP expression (Fig. 4a). This effect was sustained until adult day 5 for all glucose concentrations tested (Fig. 4b, c) and was lost at adult day 9 for the lowest concentration (1%) (Fig. 4d). We wondered if there were functional consequences of additional mitochondrial stress during adulthood on lifespan. We observed that worms grown on NGM plates and exposed to *cco-1*(RNAi) at day 1 of adulthood had lifespans similar to untreated controls (Fig 4e). We then tested animals that had been exposed to either 2% or 4% GE during development, and then exposed them to *cco-1*(RNAi) at day 1 of adulthood, and observed that lifespan was reduced compared to control animals (Fig. 4f, g). Thus, early exposure to mitochondrial stress can increase lifespan, but this is a fragile phenotype since further induction of the UPR^mt^ during adulthood results in lifespan reduction.

## DISCUSSION

Our study reveals a novel role of dietary glucose in regulating lifespan in *C. elegans*. Exposure to high dietary glucose during adulthood reduces lifespan, whereas glucose exposure restricted to developing animals has the opposite effect and extends lifespan. It is believed that mitochondrial oxidants may be responsible for mitochondria communicating to their cellular host resulting in an organism-wise shift to stress resistance and pro-longevity phenotypes [18]. High glucose concentrations are associated with a number of negative phenotypes, including decreased lifespan in *C. elegans* as well as mitochondrial dysfunction and oxidative stress in other organisms [6,19]. Consistently, impairing glucose metabolism extends lifespan in worms most likely via a ROS dependent signalling mechanism [12]. Altogether these studies paint a contradictory picture where both increased or decreased mitochondrial respiration are associated with increased lifespan. It may be that altered ROS signalling from dysregulated mitochondrial respiration triggers a hormetic response leading to increased lifespan [18].

Our data suggests that timing and duration may be an important factor in whether mitochondrial stress results in positive or negative effects on lifespan. High dietary glucose during development extends lifespan, requires UPR^mt^ genes but does induce strong activation of the UPR^mt^. However, high dietary glucose exposure during adulthood does induce an UPR^mt^ response and is associated with decreased lifespan. Thus glucose acts a stress at the cellular and mitochondrial level that limits lifespan if not alleviated during adulthood. These effects are consistent with the hyperfunction theory of aging that suggest cellular pathways activated during development can accelerate aging if activity is maintained during adulthood [20].

In contrast to other work [1], we found that glucose-mediated induction of the UPR^mt^ to extend lifespan required ROS. This suggests that dietary induced mitohormesis and UPR^mt^ may employ mechanisms different than impaired mitochondrial translation. Thus dietary mitohormesis may establish conditions in adult organisms that sensitize them to lifespan reduction via additional mitochondrial stress. Furthermore, mitochondrial stress and/or mitohormesis from dietary sources are not restricted to developmental stages in the natural world. Our data suggest that chronic engagement of the UPR^mt^ via diet sensitizes animals to additional mitochondrial stress and may be a universal lifespan-limiting factor.

## EXPERIMENTAL PROCEDURES

### Worm strains and genetics

Worms were maintained on standard NGM plates streaked with OP50 *E. coli.* In some experiments D-glucose was added to NGM plates. All strains were scored at 20°C unless indicated. Strains used in this study were obtained from the *C. elegans* Genetics Center (University of Minnesota, Minneapolis) unless indicated and include: *atfs-1(gk3094); atfs-1(et15); mev-1(kn1); isp-1(qm150); zcIs13[hsp-6::GFP]; zcIs9[hsp-60::GFP]; zcIs39[dve-1p::dve-1::GFP]* ; *zcIs19[ubl-5p::ubl-5::GFP];* zcIs14[*myo-3*::GFP(mit)] ; *ccIs4251[myo-3p::GFP(NLS)::LacZ (pSAK2)* + *myo-3p::GFP*(mit) *(pSAK4)* + *dpy-20*(+)] and *sid-1(pk3321)*.

### Fluorescence microscopy

Animals were immobilized in M9 with 5 *mM* levamisole and mounted on slides with 2% agarose pads. Animals were visualized with a Leica 6000 and a Leica DFC 480 camera. A minimum of 30 animals was scored per treatment over 3 trials. Mito-GFP animals were visualized at days 1, 5 and 9 of adulthood with a Leica TCS_SP5 confocal microscope and analyzed with LAS AF software. The mean and SEM were calculated for each trial and two-tailed t-tests were used for statistical analysis.

### Lifespan assays

Worms were grown on NGM or NGM + 1%, 2% or 4% glucose and transferred on NGM or NGM + glucose. 30-40 animals/plate by triplicates were tested at 20°C from adult day 1 until death. Worms were declared dead if they didn't respond to tactile or heat stimulus.

### RNAi experiments

RNAi-treated strains were fed with *E. coli* (HT115) containing an Empty Vector (EV)*, atfs-1* (ZC376.7)*, ubl-5* (F46F11.6), *cco-1* (F26E4.9) RNAi clones from the ORFeome RNAi library (*2*) and *dve-1*(ZK1193.5) clone from Arhinger RNAi library. RNAi experiments were performed at 20°C. Worms were grown on either NGM or NGM + 2% glucose both enriched with 1 mM Isopropyl-b-D-thiogalactopyranoside (IPTG).

### Statistics

For paralysis and stress-resistance tests, survival curves were generated and compared using the Log-rank (Mantel-Cox) test, and a 60-100 animals were tested per genotype and repeated at least three times.

## SUPPLEMENTARY FIGURES AND TABLE


