# Supplementary material for: Fragile lifespan expansion by dietary mitohormesis in C. elegans
Source: Aging (Albany NY). 2016 Jan 13;8(1):50–7. doi: 10.18632/aging.100863 (PMC4761713; doi:10.18632/aging.100863)
Supplement: Supplementary file 1 [file aging-08-050-s001.pdf]

SUPPLEMENRATY FIGURES

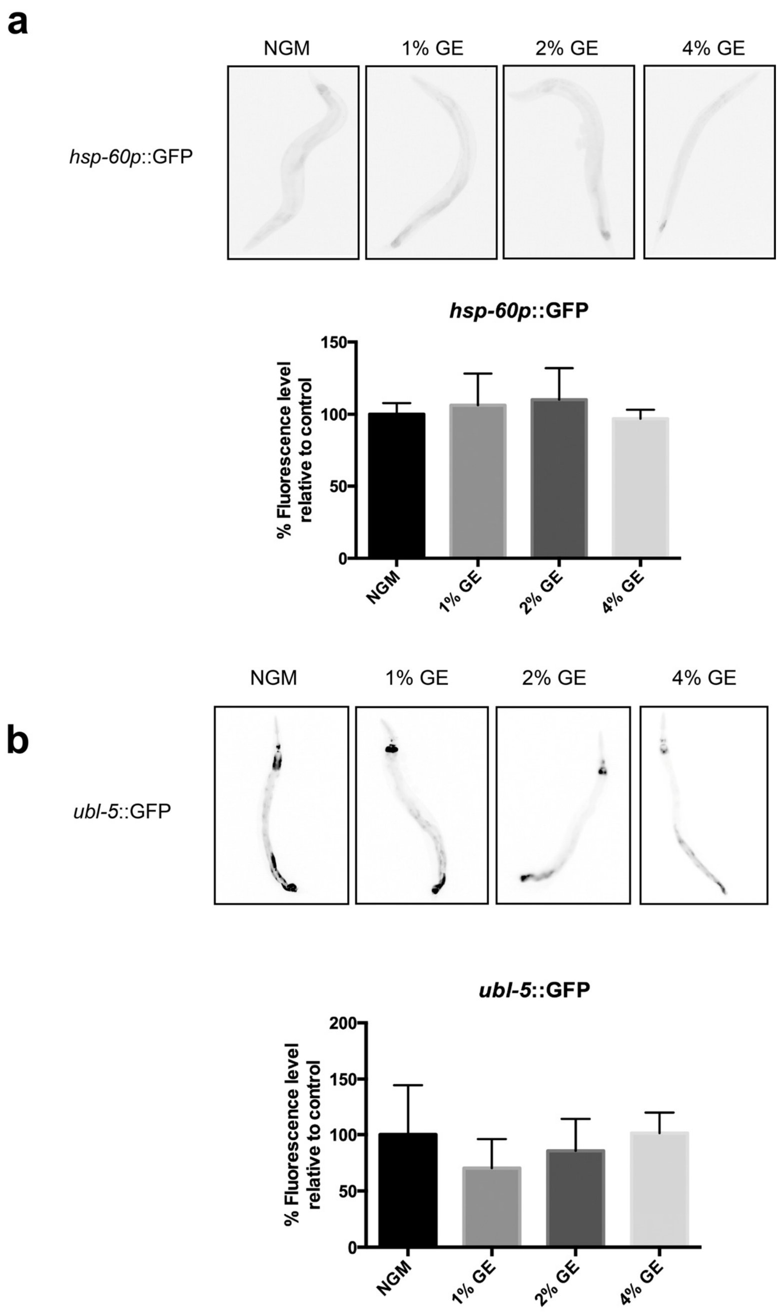

Supplementary Figure 1. GE doesn't induce HSP-60 and UBL-5 expression. (**a-b**) GE fails to induce HSP-60 expression at all GE concentration tested (mean + s.d.). (**c**) Reduction of ATFS-1 expression by RNAi abolishes GE mediated hsp-6 expression at 2% glucose (mean + s.d.).

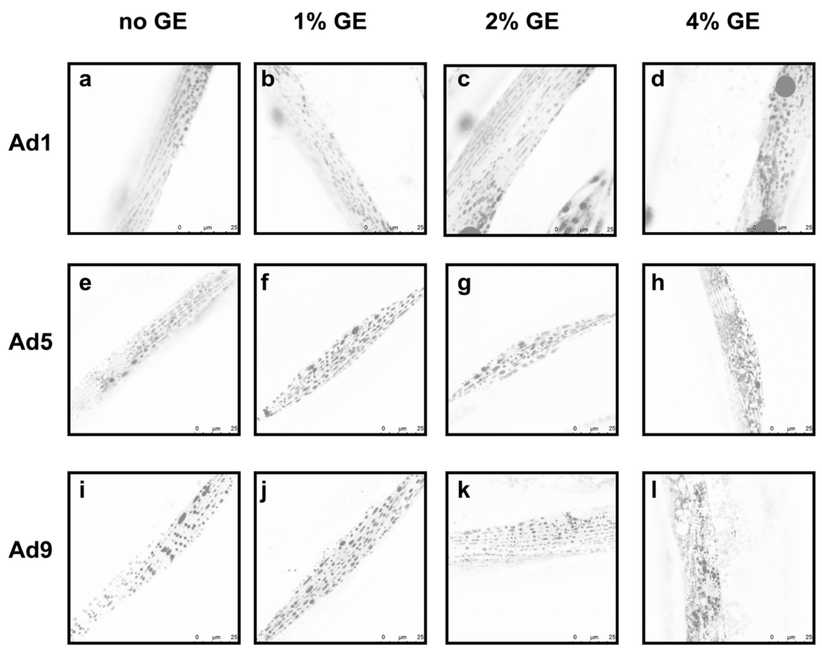

Supplementary Figure 2. Glucose and aging disrupt mitochondrial morphology. (**a**) Untreated animals show linear and uniform mitochondrial signal along muscle fibers. (**b-d)** High dietary glucose causes premature muscle and mitochondrial organization. (**e-l**) Loss of linear organization in muscle fibers is age (**e, i**) and glucose dependent (**f-h; g-l**), with more pronounced effect at 4% concentration (**l**)**.**

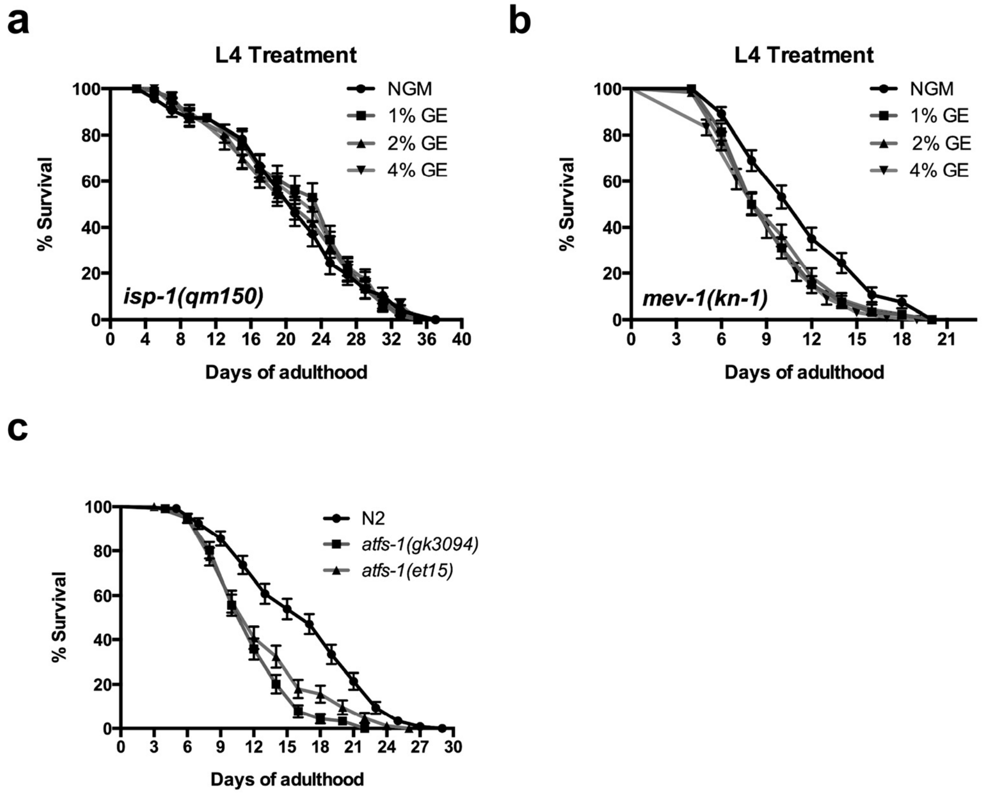

Supplementary Figure 3. Lifespan extension is dependent on functional ETC and oxidative stress. (**a-b**) GE until L4 stage failed to increase lifespan of *isp-1(qm150)* and *mev-1(kn-1)* mutants. (**c**) *atfs-1 (et15)* and *atfs-1(gk3094)* mutants have reduced lifespans compared to N2 animals (P<0.0001 compared to control).

Supplementary Table 1

Figure	Strains	Mean lifespan	pValue	75th percentile (days)	maximum lifespan	Total number of death/total
	EV	17		20	29	373/383
	+ 2% GE	17	n.s. 0.3862	18	29	404/412
	+ 4% GE	13	**<0.0001**	17,5	24	215/219
	ATFS-1 RNAi	14		19	25	357/373
	+ 2% GE	13	**0.0019**	15	25	441/449
	+ 4% GE	11	**<0.0001**	17	21	213/218
**Fig. 2**	DVE-1 RNAi	13		20	24	245/251
	+ 2% GE	11	**<0.0001**	23,5	16	290/292
	+ 4% GE	13	**0.0105**	23,5	15	103/106
	UBL-5 RNAi	14		49,5	25	185/192
	+ 2% GE	12	**<0.0001**	30	19	207/207
	+ 4% GE	13	**<0.0001**	34	15	101/106
	N2 Full life Treatment (FL) 0%	17		18	24	133/142
	N2 FL 1%	16	n.s. 0.7242	19	27	138/142
	N2 FL 2%	16	**0.0448**	18	27	112/114
	N2 FL 4%	14	**<0.0001**	15	22	123/124
	N2 L4 Treatment (L4) 0%	16		20	28	130/140
	N2 L4 1%	18	n.s. 0.6619	19	28	132/144
	N2 L4 2%	21	**<0.0001**	25	32	102/142
	N2 L4 4%	22	**<0.0001**	23	30	131/152
	N2	20		21	31	108/120
	N2 adult Treatment (Ad) 1	14	**<0.0001**	17	23	103/120
	N2 Ad 3	15	**<0.0001**	16	25	113/120
**Fig. 3**	N2 Ad 5	16	**<0.0001**	18	23	104/119
	N2 Ad 9	16	**<0.0001**	20	27	97/118
	atfs-1(et15) L4 Treatment (L4) 0%	13		18,5	28	119/145
	atfs-1(et15) L4 1%	11	**<0.0001**	15	24	105/129
	atfs-1(et15) L4 2%	9	**<0.0001**	12	24	115/144
	atfs-1(et15) L4 4%	9	**<0.0001**	12	20	117/136
	atfs-1(gk3094) L4 Treatment (L4) 0%	17		18	25	109/116
	atfs-1(gk3094) L4 1%	17	0.0016	18	25	115/119
	atfs-1(gk3094) L4 2%	17	n.s. 0.0606	20	25	115/119
**Fig. 3**	*atfs-1(gk3094)* L4 4%	16	n.s. 0.0750	20	26	109/120
	N2 L4 0%	14		17	27	102/120
	N2 L4 2%	17	**<0.0001**	20	32	101/120
	N2 L4 2% + NAC 10 mM	13	n.s. 0.8417	17	30	92/113
	N2 no GE	19		22	27	100/120
	+ *cco-1* RNAi Ad1	21	n.s. 0.2189	22	29	119/120
	+ *cco-1* RNAi Ad1	19	n.s. 0.4701	22	33	113/120
	+ *cco-1* RNAi Ad5	21	**0.0043**	24	31	120/120
	+ *cco-1* RNAi Ad9	21	**0.0238**	24	29	114/120
	N2 1% GE L4	19		24	29	106/120

**Fig. 4**	N2 no GE	19		22	27	100/120
	+ *cco-1* RNAi Ad1	21	n.s. 0.2189	22	29	119/120
	+ *cco-1* RNAi Ad1	19	n.s. 0.4701	22	33	113/120
	+ *cco-1* RNAi Ad5	21	**0.0043**	24	31	120/120
	+ *cco-1* RNAi Ad9	21	**0.0238**	24	29	114/120
	N2 1% GE L4	19		24	29	106/120
	+ *cco-1* RNAi Ad1	19	n.s. 0.7372	18	33	122/122
	+ *cco-1* RNAi Ad1	19	n.s. 0.4883	22	29	118/120
	+ *cco-1* RNAi Ad5	17	**0.0322**	22	29	111/120
	+ *cco-1* RNAi Ad9	17	n.s. 0.0871	22	31	113/120
	N2 2% GE L4	19		24	31	113/120
	+ *cco-1* RNAi Ad1	17	**0.0008**	18	31	120/120
	+ *cco-1* RNAi Ad1	19	n.s. 0.5849	24	29	116/120
	+ *cco-1* RNAi Ad5	19	n.s. 0.2594	24	29	104/120
	+ *cco-1* RNAi Ad9	17	n.s. 0.0601	22	31	118/120
	N2 4% GE L4	19		24	33	106/120
	+ *cco-1* RNAi Ad1	17	**<0.0001**	18	25	116/120
	+ *cco-1* RNAi Ad1	21	n.s. 0.7436	24	31	120/120
	+ *cco-1* RNAi Ad5	21	n.s. 0.5512	24	33	110/120
	+ *cco-1* RNAi Ad9	21	n.s. 0.9602	24	33	103/120
	N2 no GE	19		22	27	100/120
	N2 1% GE L4	19	n.s. 0.1968	24	29	106/120
	N2 2% GE L4	19	n.s. 0.0612	24	31	113/120
	N2 4% GE L4	19	**0.0057**	24	33	106/120
**Supp. Fig. 3**	*isp-1(qm150)* L4 0%	21		25	37	81/116
	isp-1(qm150) L4 1%	25	n.s. 0.9551	26	35	63/87
	isp-1(qm150) L4 2%	21	n.s. 0.8744	26	35	99/118
	isp-1(qm150) L4 4%	23	n.s. 0.6732	26	35	102/118
	mev-1(kn-1) L4 0%	12		14	20	97/119
	mev-1(kn-1) L4 1%	8	**<0.0001**	11	20	102/120
	mev-1(kn-1) L4 2%	10	**<0.0001**	11	20	101/120
	mev-1(kn-1) L4 4%	9	**<0.0001**	10	19	104/120
	N2	17		20	29	117/121
	atfs-1(gk3094)	12	**<0.0001**	13	22	99/116
	atfs-1(et15)	12	**<0.0001**	15	26	93/120
